# The Economic Burden of Malnutrition in Pregnant Women and Children under 5 Years of Age in Cambodia

**DOI:** 10.3390/nu8050292

**Published:** 2016-05-14

**Authors:** Regina Moench-Pfanner, Sok Silo, Arnaud Laillou, Frank Wieringa, Rathamony Hong, Rathavuth Hong, Etienne Poirot, Jack Bagriansky

**Affiliations:** 1 Ibn360 Pte Ltd., 3 Pickering Street, 02-36 Nankin Row, China Square Central, Singapore 048660, Singapore; 2 Office of the Council of Minister, Council of Agriculture of Rural Development, Russian Federation Blvd, Phnom Penh 12100, Cambodia; silosok@yahoo.com.au (S.S.); epoirot@unicef.org (E.P.); 3 United Nations Children’s Emergency Fund, Maternal, Newborn and Child Health and Nutrition section, no11 street 75, 12202 Phnom Penh, Cambodia; rhong@unicef.org; 4 Institute of Research for Development (IRD), UMR Nutripass IRD-UM2-UM1, 34000 Montpellier, France; franck.wieringa@ird.fr; 5 ICF International, 530 Gaither Road, Suite 500, Rockville, MD 20850, USA; rathavuth.hong@icfi.com; 6 Independent Consultant, 606 Park Lane, Decatur, GA 30033, USA; bagrianskyjack@gmail.com

**Keywords:** malnutrition, stunting, economic burden, Cambodia, Demographic Health Survey, 2014

## Abstract

Malnutrition is locked in a vicious cycle of increased mortality, poor health, impaired cognitive development, slow physical growth, reduced learning capacity, inferior performance, and ultimately lower adult work performance and productivity. The consensus of global scientific evidence indicates that lowering the rates of malnutrition will be an indispensable component of any successful program to raise the quality of human capital and resources. This study used a “consequence model” to apply the coefficient risk-deficit on economic losses, established in the global scientific literature, to Cambodian health, demographic, and economic data to develop a national estimate of the value of economic losses due to malnutrition. The impact of the indicators of malnutrition analyzed represent a burden to the national economy of Cambodia estimated at 266 million USD annually (1.7% of GDP). Stunting is reducing the Cambodian economic output by more than 120 million USD, and iodine deficiency disorders alone by 57 million USD. This economic burden is too high in view of Cambodia’s efforts to drive economic development. The government should rapidly expand a range of low-cost effective nutrition interventions to break the current cycle of increased mortality, poor health and ultimately lower work performance, productivity, and earnings.

## 1. Introduction

In the late nineties, in a regional Asian effort to advocate on the importance of malnutrition, the United Nations Children’s Emergency Fund (UNICEF) and the Asian Development bank assessed the impact of malnutrition on the economy in seven countries. It was concluded that malnutrition, with its insidious effect over the life span of a child, will cost the economies at least 3% of their gross domestic product (GDP) [1]. Malnutrition, such as micronutrient deficiencies, has been more and more associated with economic productivity losses [2,3]. The World Bank estimated that the combined economic cost of iron deficiency, iodine deficiency, and vitamin A deficiency in developing countries could waste as much as 5% of the GDP [4], while Horton and Ross estimated that iron-deficiency anemia alone could cost an average loss of 4.5% of the GDP [2]. The recently published Global Nutrition Report 2015 reported on a study estimating a reduction of 10.3% in GDP in Malawi due to malnutrition [5].

Cambodia’s expanding economy, along with rising incomes and easier access to food, is expected to contribute to the reduction of currently high rates of malnutrition, as reported in 2010 [6]. According to an economic estimation of the burden of malnutrition in Cambodia using the 2010 Cambodian Demographic Health Survey (CDHS) data led by the Council for Agricultural and Rural Development (CARD), the negative health outcomes from child malnutrition present a heavy economic burden of increased health expenditure coupled with decreased productivity and affect the rights of women and children to be healthy participants in society. The Royal Government of Cambodia, with the support of UNICEF/WFP, estimated that malnutrition has caused an annual loss of over USD 250–400 million to Cambodia’s gross domestic product [7].

The 2014 CDHS [8] shows that in nutrition, Cambodia did not meet the 2015 Cambodia Millennium Development Goal (CMDG) targets [9]. 32.4% and 23.9% of children are still respectively stunted and underweight—in comparison to the set target of 24.5% and 19.2% by 2015. Therefore, the government is currently working to expand a range of low-cost effective nutrition interventions to break the current cycle of increased mortality and poor health, impaired cognitive and physical growth, reduced learning capacity, and ultimately lower work performance, productivity, and earnings.

Regardless of the recent attention in the national policy dialogue and strategies, the nutrition agenda remains under-resourced both by the government and donors, constraining the much-needed scale-up of a life-cycle approach, particularly towards children under 5 years of age. The aim of this article was to re-examine and update the economic implications of malnutrition in Cambodia and lay the ground work for a general consensus among Government and donors on the need to invest in nutrition.

## 2. Methodology-Consequence Model

The assessment explored the economic losses associated with 14 nutrition indicators collected during the 2014 Cambodian Demographic Health Survey (a nationally representative survey) [8] via 4 discrete pathways: (i) mortality in children with consequent lost value of a future workforce (Pathway 1); (ii) child cognition deficit resulting in inferior school performance and adult productivity (Pathway 2); (iii) current value of reduced productivity in working adults (Pathway 3); and (iv) current value of excess and preventable healthcare and welfare utilization (Pathway 4). The same methodology was used in other countries, such as Laos [10], Albania [11], and in Cambodia with the 2010 Cambodian Demographic Health Survey [7]. The 14 nutrition indicators are described in Table 1 and are divided into three groups: (i) four indicators for pregnant women; (ii) eight indicators for children under 5 years of age; and (iii) two indicators for adults.

### 2.1. Pathway 1: Child Mortality Attributable to Malnutrition and Estimated Value of Workforce Lost to Child Mortality

The scale of child mortality emerging from malnutrition is hidden in a negative synergy of malnutrition, infection, disease, and premature death of children. Except for rare cases of kwashiorkor or nutritional marasmus, malnutrition is rarely listed as the cause of death. However, malnutrition is a distinct, measureable and often significant contributing factor to child mortality and is globally recognized as the underlying cause of up to 45% of all child death [17]. Estimating the national impact of malnutrition on child mortality is based on current rates of child death (35 children under 5 years of age death, 28 infant deaths and 18 neonatal deaths for 1000 live births [8]). In addition to the rates of mortality, every effort was made to ground this analysis in the specific causes of child mortality in Cambodia. Much of the evidence attributing mortality to various indicators of malnutrition was based on studies and intervention trials which established links with “all-cause mortality”. However, in some cases the literature has developed disease or infection-specific risks. Therefore, in addition to country-specific mortality rates, wherever possible, this analysis was based on the underlying causes of child mortality specific to Cambodia. Since no national data has been identified, mortality from specific infections such as diarrhea, respiratory disease, measles, and other infections is taken from WHO Child Mortality by Cause 2000–2010 [18].

This pathway applies coefficients of risk for mortality for specific maternal (anemia, body mass index (BMI) and height) and child (wasting, underweight, sub-optimal breastfeeding, vitamin A deficiency, zinc deficiency, and neural tube defects) nutrition indicators. Those relative risks used in the global scientific literature (see Table 2) have been tailored to the local Cambodian context to paint a general picture of child mortality.

For pathway 1, to estimate the number of deaths attributable to maternal and child malnutrition, the following algorithm was used [10,11]:

Number of Deaths attributed = Population attributable risk × Mortality in risk group affected

With Population attributable risk, the Population Attributable Risk (PAR) is a function of the prevalence of the nutrition indicator along with the severity of the mortality risk as expressed by the Relative Risk (RR: see Table 2). It is calculated with the following formula: (Prevalence × (RR − 1))/(1 + (Prevalence × (RR − 1))); Mortality in risk group affected, number of deaths per year based on national data

The estimated value of workforce lost to child mortality was then estimated. This value is simply derived as a lost workforce—by taking a discounted net present value (NPV) of future lost earnings. The NPV includes a delayed earnings stream that presumes entry into the workforce at an average of 15 years of age—a delay ranging from 15 years for perinatal and neonatal deaths to an average of 13 years for the 6–59 months old cohort. Furthermore, this economic perspective attributes an economic value only to the 82.6% of children who would be projected to participate in the labor force as adults. In other words, this economic calculation attributes no value at all to approximately 17% of these child deaths who are not projected to participate in the labor force and be economically active.

Net Present Value of Lost Workforce due to Child Mortality = Child Deaths Attributed to Malnutrition × Average Wage × Labor Force Participation Rate × Net Present Value (NPV) of work life with a delayed earnings’ stream

Average Earnings, $US 1,253/year; based on $US 177 per month per household and average number of workers per household estimated at 1.7; Labor Force Participation, All: 82.6%, Male: 87.9% and Female: 77.5% [23]; Net Present Value, Net Present Value (NPV) is a subjective factor used to define the value of future goods or services and expresses that value in current currency. To calculate this NPV of lost future earnings due to the various indicators of malnutrition, we used a 3% discount rate, recommended by the World Bank [24] for social investments. As a sensitivity parameter, a 7% discount rate is also used as recommended by other organizations [25].

### 2.2. Pathway 2: Child Cognition Deficit Resulting in Inferior School Performance and Adult Productivity

Children, even mildly or moderately undernourished, score poorly on tests of cognitive functions, psychomotor developments, and fine motor skills. With lower activity levels, they interact less frequently with their environment and thus fail to acquire physical and intellectual skills at normal rates. These early childhood deficits determine to a large extent the ability to capitalize on educational opportunities and later employment opportunities, resulting in an adult productivity deficit. There is substantial evidence that after correction for poverty, nutrition has independent and additive impacts on child growth, cognition, and development [26]. Pathway 2 focuses on childhood anemia, neural tube defects, and stunting—nutrition indicators strongly associated with slow growth, reduced cognition, suboptimal school performance, and reduced adult earnings. Coefficient Risk-Deficit were applied according to global scientific literature (Table 3).

The general algorithm for annual losses due to depressed future productivity (DFP), mixing global and national parameters, was applied [10,11] to each of the three nutrition indicators (stunting, iron deficiencies, and neural tube defects):

Annual Loss DFP = Number of target population with the deficit or the risk (using population from the target population and prevalence found in 2014CDHS [8]) × Average Earnings × Labor Force Participation × Average Work-Life × Coefficient Risk-Deficit × Net Present Value

For stunting, a 19.8% deficit (see Table 3) in earnings is applied for the 150 thousand stunted children who are projected to be professionally employed in government, education, and other services (26.5% of total labor force) where schooling and cognitive acuity are regarded as key components of productivity [23]. For iron deficiency anemia, the literature from child psychology, nutrition, and economic science finds that development deficits related to iron status in children less than 5 years old are associated with a 4% drop in earnings [2]. In general, studies show that iron supplementation in children aged under 5 years led to cognitive improvements which were sustained into adolescence with a correlation coefficient of 0.62 [2]. Our analysis applied a correlation coefficient of 0.62 to findings of 4% earnings’ deficit result in coefficient of 2.5% lower future earnings and productivity [2].

Finally, for iodine deficiency disorders (IDDs), substantial literature links IDD to a deficit of IQ points—which is in turn linked to earnings deficits of about 9.6%. This coefficient of deficit is then applied only to the 13% of a CDHS national sample that indicated severe iodine deficiency.

### 2.3. Pathway 3: Current Value of Reduced Productivity in Working Adults

Weakness, fatigue, and lethargy brought on by anemia result in measurable productivity deficits in manual labor. Aerobic capacity, endurance, and energy efficiency are compromised by 10%–50% [28]. Our analysis has applied the coefficients of 5% deficit among manual laborers [2] and an additional 12% loss among heavy manual laborers [2]. As with previous analyses, productivity deficits are only applied to individuals participating in the labor force; 88.8% for men and 80.4% for women [23]. In the case of anemia, we applied an additional screen to include only individuals in manual labor. The most recent Cambodia Socio-Economic Survey (CSES) found 74% of women and 72.9% of men were involved in manual labor [23]. While there is no data distinguishing normal from heavy manual labor (and the definition itself may be ambiguous), based on a global analysis done for the Copenhagen Consensus, we assumed 15% of manual labor may be classified as heavy [29].

The general algorithm for annual losses due to depressed current productivity (DCP):

Annual Loss DCP = Number of target population with the deficit or the risk (using population from the target population and prevalence found in 2014CDHS [8]) × Average Earnings × Labor Force Participation × Manual labor (%) × Coefficient Risk-Deficit (5% for manual and 17% for heavy manual)

### 2.4. Pathway 4: Current Value of Excess and Preventable Healthcare and Welfare Utilization

Multiple reviews have associated several nutrition factors to the increase or decrease of diarrhea and acute respiratory infection (ARI), the two main morbidities assessed in the Cambodia Health Monitoring Information System. In this paper, we have assessed the increase of healthcare expenditure due to zinc deficiency, none sub-optimal breastfeeding and low birth weight due to maternal nutrition status.

A wealth of literature has documented the association of suboptimal breastfeeding and increased morbidity from acute respiratory infection and diarrhea (Table 4). For example, for children 0–6 months, the most recent review in the Lancet [17] found that predominantly breastfed infants have a relative risk of 1.26 for diarrhea morbidity, whereas the risk triples for those not being breastfed at all. For zinc, Black *et al.* derive a pooled and prevalence adjusted RR of 2.85 for diarrhea and a RR of 2.07 for ARI [17]. Applying the RR of 2.85 for diarrhea to the Cambodian environment resulted in projections that seemed unreasonably high with a PAR of 50%. Therefore, we applied the low end of the confidence interval (CI) derived by Black *et al*., or RR 1.93 for diarrhea [17].

Based on the prevalence from CDHS data and distinct infection-specific risk from the literature, several Population Attributable Risks (PAR) were calculated to derive the number of cases attributable to each infection (diarrhea and ARI) and nutrition indicators. The sum of these eight individual projections gave the total excess cases to be treated at health facilities level due to none–suboptimal breastfeeding and zinc deficiency. For low birth weight, 31% of the cases were associated with maternal nutrition (data calculated during pathway 1). Finally, to estimate the cost of those excess cases, we have used the average pediatric case-costs from the 2012 Cambodia Hospital costing and financial management study [30].

## 3. Results

**Figure 1:**
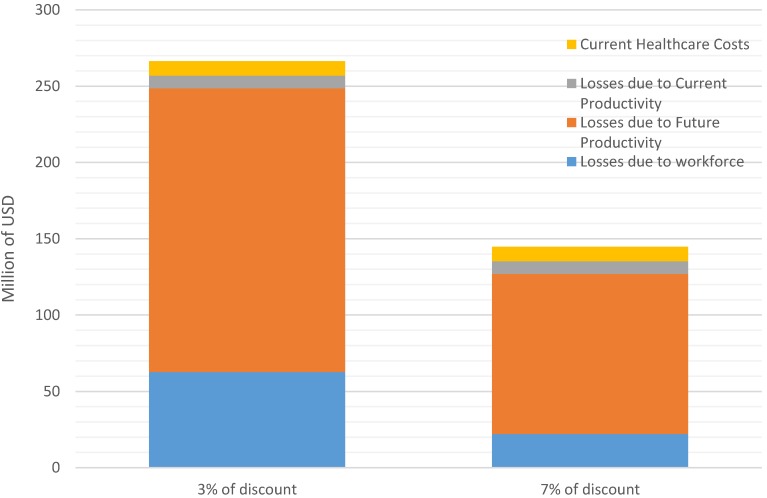
Impact of discount rates on economic burden of malnutrition.

**Figure 2:**
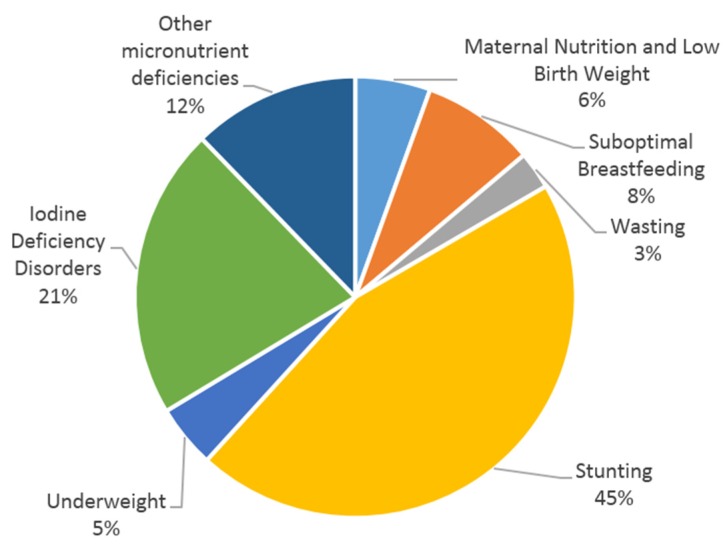
Impact on the total economic burden of malnutrition (using 3% discount rate).

The impact of the indicators of malnutrition analyzed in the report represent a burden to the national economy of Cambodia estimated at more than 260 million USD annually. This amount represents 1.7% of the estimated 2013 Gross Domestic Product (GDP) from the Ministry of Economy and Finance (15.8 billion USD). Figure 1 presents the losses from the four distinct pathways according to two internationally approved discount rates to develop the NPV (3% and 7% [24]). Applying a higher 7% discount rate shrinks the economic burden of malnutrition to about $150 million per year, representing 0.9% of GDP. According to the 2014 CDHS, more than two-thirds of the economic burden is due to stunting and iodine deficiency disorders (IDD) (Figure 2), using the discount rates 3% and 7%.

### Pathway 1: Child Mortality Attributable to Malnutrition

Maternal malnutrition and suboptimal breastfeeding along with child underweight, wasting, zinc, and vitamin A deficiencies bring a higher risk of death to children less than 5 years of age, ranging from about one-third to more than 10 times the risk of well-nourished children (Table 2). These risks, taken from global literature, together with national prevalence data from the CDHS and after statistical adjustment, are used to project 4454 thousand deaths annually, nearly one-third of all child mortality in Cambodia (estimated 13,767 dead children under 5 years of age [8]). 

During the first six months of life, non-exclusive breastfeeding is associated with about 60% of infant deaths (Table 5). As overall threats to survival subside in the 6–59 months old age group, 62% of child mortality is associated with underweight and wasting.

Presuming an entry into the workforce at average age of 15 years, the total economic losses from emerging child deaths is approximately 62 million USD (using the discount rate 3%, Table 6). About two-thirds of the loss is linked to micronutrient deficiencies, along with mother’s nutrition status and breastfeeding behavior, as opposed to traditional measurements of malnutrition such as underweight.

### Pathway 2: Reduced Future Productivity of Children

Stunting, anemia and IDD impact adult productivity by impairing children’s cognitive development through physiological changes, reducing the ability to participate in learning experiences, or both. The global literature has established that stunted children suffer a schooling deficit associated with a 19.8% decrease of adult annual income [21,26] (Table 3). This deficit is applied to stunted children who are projected to enter the service sector, where literacy, numeracy, and other intellectual skills are required. In addition, lower adult height is linked with reduced earnings in physically demanding jobs. This deficit is applied to stunted children projected to be employed in agriculture, fishing, manufacturing, and other manual jobs. Losses DCP attributed to Cambodia’s 32% prevalence of stunting total 120 million USD annually, approximately 45% of the total economic burden (Table 5). An additional 7.7 million USD are lost due to iron deficiency. Likewise, a substantial wealth of literature links IDD to a deficit of about 8 IQ points [32], which is in turn linked to earnings’ deficits of approximately 9%. Based on findings of the CDHS 2014, we project 12.8% of Cambodian children with iodine deficiency (urinary iodine concentration below 25 µg/L) [33], suggesting a future economic loss of 57 million USD annually.

### Pathway 3: Reduced Current Productivity: Iron Deficiency in Adult Workers

At current prevalence levels of iron deficiency anemia, more than 200,000 adult workers in Cambodia suffer from weakness, fatigue, and work 5%–17% less efficiently than healthy workers [2]. Therefore, annual losses DFP (based on a loss of $38.4/worker/year) add up to a national burden/loss of approximately 8.3 million USD, while at the same time, key sectors such as agriculture, fishing, manufacturing, construction, and other labor sectors are impacted—yet, those sectors build the foundation for expanding Cambodia’s economic growth

### Pathway 4: Excess Healthcare Expenditures

Malnutrition in children contributes to reduced immunity and increased infection. Consequently, malnourished children may suffer more frequently from disease or are more severely ill, which in turn translates to increased (i) utilization of health facilities and (ii) expenses for health services. Children 6–24 months of age not benefiting from adequate breastfeeding practices and children 6–59 months old with zinc deficiencies suffer 2.6 million excess preventable cases of diarrhea and respiratory illness. These cases, together with low birth weight and birth defects associated with maternal nutrition status, result in excess costs to the health system and to families of approximately $9.6 million annually (cost of NTDs for health care is equal to approximately 52,000 USD/year).

## 4. Discussion

Most of Cambodia’s children face higher than normal risks of dying before their fifth birthday. More than four thousand deaths annually—nearly one-third of all child mortality—is linked to their mother’s nutrition status and breastfeeding behavior, along with child underweight or vitamin and mineral deficiencies. The present value of this lost future workforce is estimated at more than 60 million USD annually (with 3% discount rate). 2.6 million annual cases of diarrhea and respiratory infection among children are attributed to poor breastfeeding practices and zinc deficiencies, resulting in excess costs to the health care system and individual families reaching nearly $8.6 million (with 3% discount rate). Finally, children with less than normal height, iron deficiency or iodine deficiencies suffer deficits in mental and physical development, perform less well in school and consequently, will be less productive as adults, reducing GDP by 186 million USD per year (with 3% discount rate).

In total, according to the 2014 CDHS [8] and depending on the discount rate, 0.9%–1.7% of the GDP (145–266 million USD) is and will be lost every year due to malnutrition indicators if not tackled. This is below estimations made in other countries such as Laos in Asia (2.4% of the GDP) [10] and Malawi in Africa (above 10% of GDP) [5].

The findings of our assessment represent an improvement of 100 million USD less lost in comparison to the previous calculation done with indicators from the 2010 CDHS [6,7]. One of the reasons is due to the use of iron deficiency to estimate the economic loss of productivity instead of anemia. It has been assumed that iron deficiency was one of the principal causes for anemia [34], but recent data have shown that it is not as straight-forward [23]. A recent published paper in 2015 showed that genetic hemoglobin disorders, rather than iron deficiency, are a major predictor of hemoglobin concentration in women aged 18–45 years in Prey Veng, Cambodia [35]. The 2014 CDHS is also finding high levels of hemo-globinopathy with only 22% of the children and 47% of the women having a “normal hemoglobin” (hemoglobin A1 > 95%). Therefore, in 2010 there might have been an overestimation of the economic burden of anemia due to iron deficiency. However, the loss of productivity because of anemia remains. The anemia prevalence has not declined from 2010 to 2014 [6,8], but most of it has no relationship with nutrition deficiencies.

Most of the projected losses (45%) are linked to stunting. Similar findings were observed in Albania, where stunting represented also approximately 50% of the economic losses [11]. Childhood stunting is one of the most significant obstacles to human development, as it is a well-established risk marker of poor child development [36]. If Cambodia follows the 40% goal reduction in stunting, set forward for 2025 by the World Health Assembly [36], the government could prevent a yearly loss of approximately 50 million USD. To do so, many complementary strategies to improve nutrition need to be considered, including: (i) reduce wasting [37]; (ii) reduce low birth weight; and (iii) increase appropriate feeding practices during pregnancy and in children under 5 years of age [38,39]. It is also important to address relevant agriculture and health related issues, but also water, sanitation, and hygiene (WASH) in a more comprehensive and integrated way. which makes the prevention of stunting more complex. For example, Cambodia is still among countries with a high prevalence of the population without access to adequate sanitation (44%) [8], which could negatively impact all efforts made towards stunting reduction if WASH interventions are not implemented.

Even if in Cambodia, less than 3% of the 266 million USD in annual economic burden is associated with acute malnutrition requiring in-patient or out-patient clinical life-saving medical care, it is essential to continue the efforts to treat those affected children. Evidence suggests episodes of wasting negatively affect linear growth and, therefore, undermine child growth and development [37]. During a workshop in 2014, experts agreed that wasting and stunting share many common risk factors and therefore clearer policy directives are needed to encourage and facilitate practical links for more integrated programming [40].

As highlighted in Figure 1, the second biggest cause of the economic burden is due to iodine deficiency disorders, and several interventions have been implemented in Cambodia over the last decade. Since the 2003 Government’s Sub-Decree No. 69 on mandatory iodization of salt and several related regulations, Cambodia’s supply of iodized salt has increased greatly to reach approximately 70 per cent of households around 5 years ago [41]. Unfortunately, to date, the current levels of iodine added to tested salt are unsatisfactory as 92% of those salts do not meet the government’s iodization requirements (99.6% of the coarse salt and 82.4% of the fine salt) [41]. This inappropriate iodization has a direct causality with the drastic reduction of urinary iodine concentration observed over the last few years [33]. A minimum investment of 750,000 USD/year in Cambodia (0.05 USD/person/year) could resolve the problem, as already observed during a national assessment of the National program 2011 [42]. This intervention, coupled with the implementation of an effective regulatory monitoring and enforcement system, should allow sustainable prevention of any iodine deficiency disorder. The other child micronutrient deficiencies (zinc, vitamin A, and iron) could be prevented with the development of a social marketing strategy for micronutrient supplements or food supplements to improve the quality of complementary feeding. Cambodia could use the Scaling Up Nutrition (SUN) initiative, and especially the SUN business network, to ensure that commitment from the private sector to support the national initiative to iodize all salt used at the household level and during the production of processed foods, such as fish or soya sauces, is happening.

As highlighted in the recent global nutrition report [5], scaling up nutrition-specific interventions to 90% coverage will generate a median benefit–cost ratio of 16:1 in countries like Cambodia. Therefore, investing 40 million USD (15% of the total economic burden of malnutrition in Cambodia) as described in the “Ministry of Health Conceptual budget for Cambodia’s fast track road map for improving nutrition” is recognized to be highly effective [43].

*Limitations to the conceptual model*: Converting indicators of malnutrition to an economic activity and attaching a monetary value to that economic activity could be interpreted and analyzed in many different ways. First, monetizing the consequences of malnutrition is dependent on evolving evidence bases, complex methodologies and national health, demographic and economic statistics of uneven quality; Second, many factors beyond individual physical and intellectual potential determine earnings or work performance. Workplace incentives, available technology, and sense of opportunity all affect how increased human potential translates into actual improved productivity. In addition, productivity growth was not considered in the model, even if we can speculate that today’s children are likely to be much more productive in their working life than their parents are today; Finally, benefits of improved nutrition extend beyond the workplace to a range of “voluntary” activities, including parenting and household activities to educational improvement, entrepreneurial pursuits, and community participation. In a world where improvement in nutrition, health, and subsequent productivity will emerge mainly from individual choices and behaviors, the significance of these “voluntary” activities cannot be overstated. For all these reasons and more, the margin of error is large and the calculations should be considered as an order of magnitude. These are projections to focus and facilitate policy discussion and present a solid and conservative case for policy discussion. Therefore, data judgments or assumptions are consciously and consistently “biased” to minimize the impact of malnutrition. Consequently, conclusions drawn may be considered conservative low-end estimates.

## 5. Conclusions

This article used a proven methodology on how to calculate the economic burden of malnutrition in regard to 14 key nutrition indicators for Cambodia. It showed that Cambodia is losing approximately 266 million USD per year by not addressing malnutrition, in particularly stunting and iodine deficiency. The study does not suggest easy wins for addressing those two nutrition problems, but it suggests and urges the government of Cambodia to undertake a thorough review of its current policies and directives to assess if they address malnutrition in the most optimal way. Cambodia’s efforts to expand its economic growth is hindered by a sub-optimal labor force and high avoidable costs for the health care system. It is key to reach out and link with other sectors—*i.e.*, education, sanitation, and poverty reduction schemes—to address malnutrition and to attract further investment so that nutrition specific and nutrition sensitive programs can be implemented in the most synergetic and optimal way. Only through this joint effort and focus can Cambodia further thrive and prepare a healthy population and labor force.

## Figures and Tables

**Table 1:** Fourteen Nutrition Indicators: Cases and Risk Groups Derived from Demographic Health Survey 2014, Cambodia [8].

Risk Group	Nutrition Indicator	Prevalence	Cases or at Risk (000)	Risk or Deficit
393 thousand Pregnant Women	Low Body Mass Index	14%	55.1	Infant and Maternal Mortality
	Short Stature	5%	21.2	
	Anemia	53%	209.3	
	Annual projected Neural Tube Defects (NTDs) due to folate deficiency ^a^	-	0.722	
1.6 Million Children <5 Years of Age	Suboptimal Breastfeeding	35%	273.8	Mortality and Morbidity
	Low Weight for Height	10%	137.1	Mortality
	Low Weight for Age	24%	341.3	
	Low Height for Age	32.4%	462.6	Growth, Development and Productivity
	Vitamin A Deficiency ^b^	9%	124.2	Mortality
	Zinc Deficiency ^c^	67.5%	963.8	Mortality and Morbidity
	Iodine deficiency ^d^	12.8%	182.8	Growth, Development and Productivity
	Iron deficiency ^e^: Childhood	7%	104.2	Growth, Development and Productivity
10 Million Adults 15–64 years	Iron deficiency: Women	2.9%	159.7	Strength, Endurance and Productivity
	Iron deficiency: Men	1.1% ^f^	55.6	

Note: ^a^ Estimating an annual NTD of 2.55/1000 birth; number of annual birth: 393,336; 72% of NTDs related to folic acid; ^b^ Retinol binding protein below 0.70 µmol/L [12]; ^c^ Zinc deficiency was defined as below 9.9 µmol/L [13]; ^d^ Urinary iodine Concentration is associated with large intra-individual variation. During the national survey, urine was collected only one time while it is recommended to collect it in a repeated manner. Therefore, we preferred to use the prevalence of severe deficiency to prevent overestimation of the losses [14]; therefore, urinary iodine concentration below 20 µg/L was used [15]; ^e^ Ferritin below 12 µg/L for children 6–59 months and below 15 µg/L for women [12]; ^f^ Based on ratio of male to female anemia found in Vietnam survey [16].

**Table 2:** Relative risk of mortality.

	**Relative Risk**			
**Child Mortality Associated to Maternal Nutrition Status**				
anemia [19]	1.25			
BMI < 18.5 [17]	1.71			
Height < 145cm [17]	2.2			
**Neonatal Mortality Attributed to Low Birth Weight**				
2000–2499 g [17]	2.8			
<2000 g [17]	8.1			
**Post Neonatal Mortality Attributed to Low Birth Weight**				
Post Neonatal Mortality [20]	1.98			
**Mortality Associated with Severe, Moderate, and Mild Wasting and Underweight** [17,18,21]				
	***Wasting***	***Underweight***		
Pneumonia among children with anthropometric indicator *z*-score < −3SD	9.7	10.1		
Pneumonia among children with anthropometric indicator −3SD < *z*-score < −2SD	4.7	3.1		
Pneumonia among children with anthropometric indicator −2SD < *z*-score < −1SD	1.9	1.9		
Diarrhea among children with anthropometric indicator *z*-score < −3SD	12.3	11.6		
Diarrhea among children with anthropometric indicator −3SD < *z*-score < −2SD	3.4	2.9		
Diarrhea among children with anthropometric indicator −2SD < *z*-score < −1SD	1.6	1.7		
Measles among children with anthropometric indicator *z*-score < −3SD	9.6	7.7		
Measles among children with anthropometric indicator −3SD < *z*-score < −2SD	2.6	3.1		
Measles among children with anthropometric indicator −2SD < *z*-score < −1SD	1	1		
Other among children with anthropometric indicator *z*-score < −3SD	11.2	8.3		
Other among children with anthropometric indicator −3SD < *z*-score < −2SD	2.7	1.6		
Other among children with anthropometric indicator −2SD < *z*-score < −1SD	1.7	1.5		
**Infant Mortality by Breastfeeding Behavior** [22]				
	***0–6 Months***			***6–24 Months***
	***Predominant***	***Partial***	***None***	***None***
Diarrhea	2.28	4.62	10.53	2.1
Pneumonia	1.75	2.49	15.13	1.92
All Mortality	1.48	2.85	14.4	3.68
**Diarrhea and measles mortality associated with vitamin A deficiency** [17]				
Diarrhea and measles mortality	1.32			
**Mortality associated to zinc deficiency** [17]				
ARI	1.96			
Diarrhea	2.01			
**Mortality Associated to Neural Tube Defects (NTD), Estimated with United States Centers for Disease Control and Prevention**				
NTDs	80% of 2 births out of 1000 births			

Note: SD (Standard deviation), BMI (Body Mass Index), ARI (Acute Respiratory Infection).

**Table 3:** Coefficient risk-deficit on economic losses.

	**Coefficient Risk-Deficit**
**Economic Loss from Stunting**	
stunting and productivity at school [26]	19.80%
moderate stunting and direct earning [27]	6.04%
severe stunting and direct earning [27]	8.63%
**Economic Loss from Childhood Iron Deficiency**	
childhood iron deficiency [2]	2.50%
**Economic Loss from Iodine Deficiency Disorders**	
childhood iodine deficiency disorders	9.6% ^¥^
**Economic Loss from Neural Tube Defects ^†^**	
Moderate disability	50%
Severe disability	100%
**Economic Loss from Adult Iron Deficiency**	
Manual labor	5%
Heavy manual labor	17%

Note: ^†^ Given the lack of information, personal estimation has been made, ^¥^ Jack Bagriansky/UNICEF personal communication and an Indian study [14].

**Table 4:** Relative risk (RR) of Diarrhea and ARI by Breastfeeding Status [17].

Breastfeeding Behavior and Age Segment	Diarrhea Cases	ARI Cases
	RR	RR
**0–6 Months**		
None	2.65	2.48
Partial	1.68	2.07
Predominant	1.26	1.79
**6–23 Months**		
None	2.07	1.17

**Table 5:** Mortality among Children under 5 Years of Age associated with Key Indicators of Malnutrition.

Total Estimated Attributable Death		
	Death	% Risk Group/Age
**Neonatal**		
Mother’s Nutrition Status	307	4%
Mother’s NTD due to folate deficiency	540	8%
Sub Optimal Breast Feeding	527	7%
Total	**1374**	**19%**
**Infant: 1–5 Months**		
Mother’s Nutrition Status	74	3%
Sub-Optimal Breastfeeding	779	33%
Total	**853**	**36%**
**Infant: 6–59 Months**		
Partial breastfeeding (6–24 mouths)	193	4%
Wasting (WHZ < −2 *z*-score)	515	12%
Underweight (WAZ < −2 *z*-score)	859	20%
Vitamin A deficiency	91	2%
Zinc deficiency	570	13%
Total	**2227**	**51%**
**Total <5 years**	**4454**	

Note: number of death adjusted for multiple risks: Based on the individual PAR of mortality calculated for each indicator, the algorithm develops a “hybrid” PAR, statistically adjusting for multiple risks [31]. WHZ (weight for height *z*-score); WAZ (weight for age *z*-score).

**Table 6:** Summary Economic Consequences for all Indicators’ Net Present Value at 3% (Adjusted for Multiple Risks in million USD).

	Lost Workforce Due to Mortality	Lost Child Productivity	Current Lost Productivity	Current Health Costs	Total	
	Millions USD/Year	Millions USD/Year	Millions USD/Year	Millions USD/Year	Millions USD/Year	%
Maternal Nutrition	$12.9	$0.99	-	$0.8	$14.7	5.5%
Suboptimal Breastfeeding	$20.7	-	-	$1.5	$22.2	8.3%
Low Height for Weight (WHZ)	$7.4	-	-	-	$7.4	2.8%
Low Weight for Age (WAZ)	$12.3	-	-	-	$12.3	45.1%
Low Height for Age (HAZ)	-	$120.3	-	-	$120.3	4.6%
Iodine Deficiency (IDD)	-	$57.0	-	-	$57.0	21.4%
Zinc Deficiency	$8.1	-	-	$7.1	$15.3	5.7%
Vitamin A Deficiency	$1.3	-	-	-	$1.3	0.5%
Child Iron Deficiency	-	$7.7	-	-	$7.7	2.9%
Adults Iron Deficiency	-	-	$8.3	-	$8.3	3.1%
**Annual Total**	**$62.7**	**$185.9**	**$8.3**	**$9.5**	**$266**	100%

## Supplementary Materials

The following are available online at http://www.mdpi.com/2072-6643/8/5/292/s1.
